# A Moderate-Fat Diet with One Avocado per Day Increases Plasma Antioxidants and Decreases the Oxidation of Small, Dense LDL in Adults with Overweight and Obesity: A Randomized Controlled Trial

**DOI:** 10.1093/jn/nxz231

**Published:** 2019-10-14

**Authors:** Li Wang, Ling Tao, Lei Hao, Todd H Stanley, Kuan-Hsun Huang, Joshua D Lambert, Penny M Kris-Etherton

**Affiliations:** 1 Department of Nutritional Sciences, Pennsylvania State University, University Park, PA, USA; 2 Department of Food Science, Pennsylvania State University, University Park, PA, USA

**Keywords:** avocado, CVD, lipid, lipoprotein, antioxidants, MUFA, LDL oxidation, small dense LDL, lipid transfer proteins

## Abstract

**Background:**

Avocados are a nutrient-dense source of MUFAs and are rich in antioxidants. Avocados have an additional LDL cholesterol (LDL-C) lowering effect beyond that observed when their MUFAs are substituted for SFAs, especially on small, dense LDL (sdLDL) particles, which are susceptible to in vivo oxidation and associated with increased risk of cardiovascular disease (CVD).

**Objectives:**

We investigated whether a healthy diet with 1 avocado daily decreased the following secondary outcomes: circulating oxidized LDL (oxLDL) and related oxidative stress markers.

**Methods:**

A randomized, crossover, controlled feeding trial was conducted with 45 men and women, aged 21–70 y, with overweight or obesity and elevated LDL-C (25th–90th percentile). Three cholesterol-lowering diets were provided (5 wk each) in random sequences: a lower-fat (LF) diet (24% calories from fat—7% SFAs, 11% MUFAs, 6% PUFAs) and 2 moderate-fat (MF) diets (34% calories from fat—6% SFAs, 17% MUFAs, 9% PUFAs): the avocado (AV) diet included 1 Hass avocado (∼136 g) per day, and the MF diet used high oleic acid oils to match the fatty acid profile of 1 avocado. A general linear mixed model was used to analyze the treatment effects.

**Results:**

Compared with baseline, the AV diet significantly decreased circulating oxLDL (−7.0 U/L, –8.8%, *P *= 0.0004) and increased plasma lutein concentration (19.6 nmol/L, 68.7%, *P* < 0.0001), and both changes differed significantly from that after the MF and LF diets (*P* ≤ 0.05). The change in oxLDL caused by the AV diet was significantly correlated with the changes in the number of sdLDL particles (*r *= 0.32, *P *= 0.0002) but not large, buoyant LDL particles.

**Conclusions:**

One avocado a day in a heart-healthy diet decreased oxLDL in adults with overweight and obesity, and the effect was associated with the reduction in sdLDL. This trial was registered at http://www.clinicaltrials.gov as NCT01235832.

## Introduction

Oxidative modification of LDL particles plays an important role in the pathogenesis of atherosclerosis. Oxidized LDL (oxLDL) is taken up by macrophages through the upregulated scavenger receptor, leading to substantial cholesterol accumulation and foam cell formation (1, 2). Several longitudinal studies have shown that high plasma oxLDL concentration is an independent risk factor for cardiovascular disease (CVD) (3, 4). Plasma antioxidant concentrations also are an indicator of antioxidant status and have been inversely associated with CVD risk (5). Dietary antioxidant vitamins, polyphenols, and other bioactive compounds from foods (e.g., fruits, vegetables, and nuts) have been a focus of nutrition research because of their role in lowering oxidative stress and protecting LDL from oxidation (6–8).

Avocados are high in MUFAs and also are a rich source of antioxidants and polyphenols. However, their antioxidant effects have not been studied as much as those of vegetables, fruits, nuts, and the Mediterranean diet (7–10). We previously showed that the inclusion of 1 avocado per day as part of a moderate-fat, cholesterol-lowering diet had additional benefits on lowering small, dense LDL (sdLDL) and lipoprotein remnants in overweight and obese adults compared with a lower-fat diet, as well as a macronutrient- and fatty acid–matched diet (11). sdLDL particles have greater propensity for transport into the subendothelial space, increased binding to arterial proteoglycans, and susceptibility to oxidative modification (12). Accumulated oxLDL induces endothelial cell activation and the expression of many proinflammatory genes, including the endothelial cell monocyte chemoattractant protein 1 (MCP1), the vascular cell adhesion molecule 1 (VCAM1), and intercellular adhesion molecule 1 (ICAM1) (13). However, it remains unclear how avocados alter the lipoprotein metabolic pathways and their effects on LDL oxidation and proinflammatory gene expression.

The objective of this study was to determine whether the consumption of avocados as part of a healthy moderate-fat diet would lower oxLDL concentrations compared with the average American diet and 2 other cholesterol-lowering diets. Furthermore, the effects of avocados on lipid transfer proteins, plasma antioxidants, oxidative stress, and proinflammatory gene expression were also studied as secondary outcomes of our previous study (11).

## Methods

### Participants and study design

A randomized, crossover, controlled feeding trial was conducted with 45 adults with overweight and obesity (21–70 years of age, BMI 25–35 kg/m^2^, males: *n *= 27, females: *n *= 18) with LDL cholesterol (LDL-C) in the 25th–90th percentile (NHANES 2009–2010; 105–194 mg/dL for males; 98–190 mg/dL for females) (14) and normal blood pressure or well-controlled blood pressure (with medications). The recruitment process and detailed participant baseline characteristics (including BMI, blood pressure, glucose, insulin, and lipid parameters) have been reported previously (11), and the CONSORT flow diagram is presented in **Supplemental Figure 1**. The Institutional Review Board at the Pennsylvania State University approved the experimental protocol, and all participants signed a written informed consent. This study is registered at http://www.clinicaltrials.gov as NCT01235832.

**FIGURE 1:**
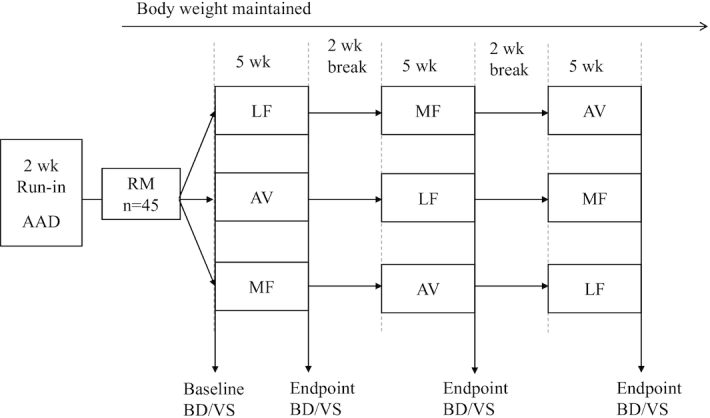
Study design: a randomized, crossover, controlled trial to evaluate the effect of a moderate-fat diet including 1 avocado per day on lipids and lipoproteins in healthy adults with overweight and obesity (aged 21–70 y). AAD, average American diet; AV, avocado diet; BD/VS, clinical visit—fasting blood draw and vital signs; LF, lower-fat diet; MF, moderate-fat diet; RM, randomization for diet treatment sequence (3 of the 6 randomized diet sequences are shown in the diagram).

A 2-wk “run-in” average American diet (AAD: 34% fat, 51% carbohydrate, 16% protein) was provided to participants before they were randomly assigned to a treatment sequence of 3 diets (5 wk each) with a 2-wk compliance break between diet periods. The 3 cholesterol-lowering diets (6–7% SFAs) were a lower-fat (LF) diet (24% fat, 59% carbohydrate, 16% protein) and 2 moderate-fat (MF) diets (34% fat, 49% carbohydrate, 16% protein) that provided similar foods and were matched for macronutrients and fatty acids. The 2 MF diets were an avocado (AV) diet that included 1 fresh Hass avocado (∼136 g fruit pulp, ∼13 g MUFAs) per day and an MF diet that mainly used high oleic acid oils to match the fatty acid content of 1 avocado. The 3 experimental diets were designed by replacing 6–7% of energy from SFAs in the AAD with carbohydrates (from grains in the LF diet) or MUFA (from 1 avocado in the AV diet or high oleic acid oils in the MF diet). The nutrient composition of macronutrients and carotenoids and tocopherols (analyzed by Food Processor SQL) of the 4 diets is shown in **Table 1**, and the major nutrient profile of 1 avocado is shown in **Supplemental Table 1**. All 3 diets met the 2010 food-based Dietary Guidelines for Americans (15), and 6-d rotating menus were developed for the diet treatments. A detailed food group distribution and a sample menu of the 3 diets have been presented previously (11). Participants’ body weight and usual physical activity level were maintained throughout the study. Fasting blood samples were obtained during the clinical visits at the end of the run-in period and the end of each experimental diet period. Details of the study protocol have been described previously (11), and a summary of the study design is shown in **Figure 1**.

**TABLE 1:** Nutrient composition of the study diets to evaluate the effect of a moderate-fat diet including 1 avocado per day on lipids and lipoproteins in healthy adults with overweight and obesity^1^

Nutrient	AAD	LF	MF	AV
Total fat, % of energy	34	24	34	34
SFA	13	7	6	6
MUFA	12	11	17	17
PUFA	7	6	9	9
Carbohydrate, % of energy	∼51	∼59	∼49	∼49
Protein, % of energy	16	16–17	16–17	16–17
Fiber, g	17	25	26	35
Cholesterol, mg	336	<200	<200	<200
Retinol, μg	579	621	506	430
α-Carotene, μg	634	1207	1495	1528
β-Carotene, μg	2078	5813	6305	6370
α-Tocopherol, μg	6	9	16	15
Lutein and zeaxanthin, μg	717	1510	2021	2393
Lycopene, μg	159	281	1271	1271

1 Nutrient content was analyzed for all menus of the 4 diets on 2100 kcal/d using Food Processor SQL software (ESHA Research) based on USDA Food Composition Databases. AAD, average American diet; AV, avocado diet; LF, lower-fat diet; MF, moderate-fat diet.

### Study measurements

#### Oxidative biomarkers: oxLDL and F_2α_-isoprostane

Plasma oxLDL concentrations were measured by ELISA using the Mercodia oxLDL ELISA kit (Mercodia Inc). The assay is based on the direct sandwich technique in which 2 monoclonal antibodies are directed against separate antigenic determinants on the oxidized apoB molecule (16, 17). The detection limit was 0.6 mU/L. Plasma F_2α_-isoprostane (8-iso prostaglandin F_2α_) was measured using Cayman's 8-isoprostane EIA kit (Cayman Chemical). The EIA displays an IC_50_ (50% B/B_0_) of ∼10 pg/mL and a detection limit (50% B/B_0_) of ∼2.7 pg/mL. The assay is based on the competition between 8-isoprostane and an 8-isoprostane acetylcholinesterase conjugate (8-isoprostane tracer) for a limited number of 8-isoprostane-specific rabbit antiserum binding sites (18). The intra- and interassay CVs of both assays were <5%.

#### Plasma antioxidants and fat-soluble vitamins

Tocopherols (α, δ, γ), carotene (α, β), lutein, and retinol were analyzed using an HPLC system consisting of 2 Shimadzu LC-20AD pumps, a Shimadzu SIL-20AC refrigerated autosampler, and an ESA 5500 Coulochem electrode array system. The potentials of the Coulochem electrode array system were set at 200, 300, 500, and 700 mV. The following commercial standards purchased from Sigma Aldrich were used for the HPLC analyses: α-, δ-, and γ-tocopherol; β-carotene; and retinol. The lutein standard was purchased from Quality Phytochemicals, and α-carotene was purchased from Chromadex. Compounds were identified by retention time and compared with pure standards (purity ≥95%). HPLC peak areas were calculated using CoulArray software (Thermo Fisher Scientific) and were converted to plasma concentrations based on external standard curve regression analyses. The linear regression analysis was carried out using GraphPad Prism (GraphPad Software).

#### Lipid transfer protein activity

The activities of plasma cholesteryl ester transfer protein (CETP), lecithin/cholesterol acyltransferase (LCAT), and phospholipid transfer protein (PLTP) were measured using homogeneous, fluorometric assay kits (Roar Biomedical). The intra- and interassay CVs were <3%.

#### Expression of proinflammatory genes by peripheral blood mononuclear cells

##### Peripheral blood mononuclear cell isolation

Blood was collected directly into cell preparation tubes containing sodium citrate and Ficoll Hypaque density fluid (BD Vacutainer CPT) and immediately centrifuged for 30 minutes (1800 × *g*, 4°C for 30 min). The peripheral blood mononuclear cell (PBMC) layer was removed, washed with PBS, and centrifuged (900 × *g*, 15°C for 15 min). After 2 washes in PBS, the cells were resuspended in 100 μL TRIzol reagent and then stored at −80°C for analysis at the end of the clinical trial.

##### Total RNA extraction and reverse transcription

Total RNA was isolated from the PBMC samples using TRIzol reagent according to the manufacturer's instructions (Life Technologies). The concentration of total RNA was determined by UV absorbance spectrophotometry (NanoDrop ND-1000). The reverse transcription reaction was performed in a total volume of 20 μL and consisted of 4 μL 5 × M-MLV RT buffer, 250 μmol/L dNTP mix, 50 ng oligo-dT15 primer, 20 U ribonuclease inhibitor, and 200 U M-MLV reverse transcriptase (Promega). The reaction was performed at 42°C for 30 min, 37°C for 30 min, and 94°C for 5 min.

##### Quantitative real-time PCR

Four target genes related to oxLDL-triggered monocyte recruitment and inflammatory response were selected for RT-PCR analysis on a subset of randomly selected subjects (*n *= 21): *MCP1*, *VCAM1*, *ICAM1*, and interleukin 1 beta (*IL1B*). The primers were made by the Nucleic Acid Facility, Penn State University (University Park, PA). Gene primer sequences are listed in **Supplemental Table 2**. The iQ SYBR Green Supermix (Bio-Rad Laboratories) was used for RT-PCR. The reaction was conducted in a total volume of 20 μL in 96-well plates. The PCR reaction conditions for each cycle were as follows: 94°C for 5 min, followed by 40 cycles at 94°C for 30 s, 60°C for 30 s, and 72°C for 30 s. The 2^−ΔΔCt^ method was used to determine relative levels of gene expression (19). Final results are expressed as fold change, using GUSB (glucuronidase, lysosomal exoglycosidase) as the endogenous control gene and comparing the end points after each diet period to the baseline.

### Statistical analysis

**FIGURE 2:**
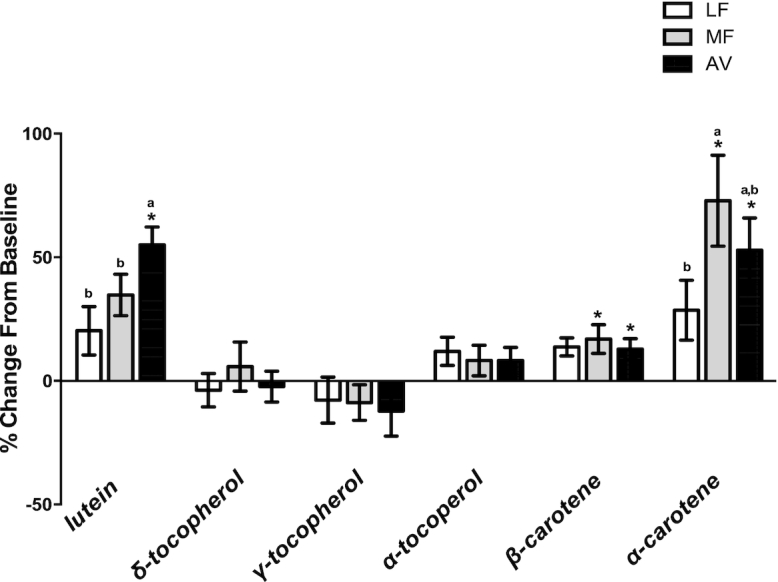
The percentage change of plasma antioxidants after 5 wk of consumption of LF, MF, and AV diets compared with baseline in healthy adults with overweight and obesity (aged 21–70 y). All values are means ± SEMs (*n *= 42–45). *The concentrations are significantly higher compared with the baseline (*P* < 0.05). Labeled means without a common letter differ, *P* < 0.05. Statistical models were run on the raw change values. AV, avocado diet; LF, lower-fat diet; MF, moderate-fat diet.

**FIGURE 3:**
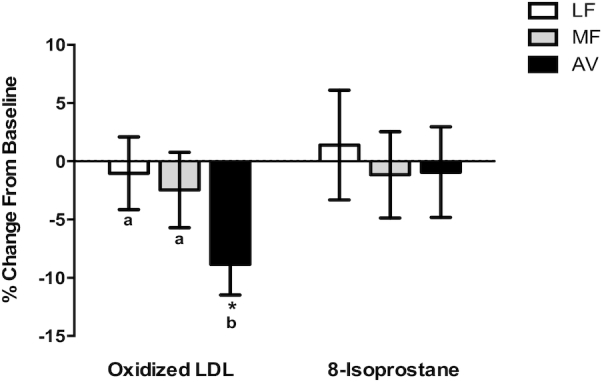
The percentage change of plasma oxidative markers after 5 wk of consumption of LF, MF, and AV diets compared with baseline in healthy adults with overweight and obesity (aged 21–70 y). All values are means ± SEMs (*n *= 42–45). *Values are significantly different from the baseline (*P* < 0.05). Labeled means without a common letter differ, *P*  < 0.05. Statistical models were run on the raw change values. The percentages of changes from baseline are shown for visualization. AV, avocado diet; LF, lower-fat diet; MF, moderate-fat diet.

Statistical analyses were performed with SAS (version 9.2; SAS Institute). The mixed-models procedure (PROC MIXED) was used comparing the effects of the 3 diets on the change value (from baseline) of all outcome variables. Potential carryover effects were assessed by including diet sequence, period, and diet-by-period interaction as a fixed effect in the model; age, BMI, sex, and diet-by-sex interaction were included as covariates. The Shapiro-Wilk test was used to assess normality of residuals in the mixed model. Tukey post hoc test was used to adjust for multiple comparisons of 3 diets. Correlations between plasma antioxidants and lipoprotein end points were determined using Pearson correlation coefficient analysis. Fisher Z-transformation was used to compare the correlations among different diets. Statistical models were run on the raw change values; the percentages of changes from baseline are shown in **Figures 2** and **3**. Three extreme values were removed from the calculation of average percentage changes in Figure 2.

## Results

### Plasma concentrations of fat-soluble antioxidants and vitamins

Plasma concentrations of retinol, lutein, α-carotene, β-carotene, α-tocopherol, γ-tocopherol, and δ-tocopherol are shown in **Table 2**. The percentage changes of these antioxidants following consumption of the LF, MF, and AV diets from baseline are shown in Figure 2. Treatment effects were seen only for lutein and α-carotene. Compared with baseline, only the AV diet increased plasma lutein (19.6 nmol/L, 68.7% *, P* < 0.0001), and the change was greater (*P *= 0.0005 and *P *= 0.008) than that of the LF (1.1 nmol/L, *P *= 0.7) and MF (7.0 nmol/L, *P *= 0.1) diets. Both MF and AV diets increased α-carotene (MF: 30.2 nmol/L, *P* < 0.001; AV: 17.2 nmol/L, *P *= 0.03) and β-carotene from baseline (MF: 21.4 nmol/L, *P *= 0.01; AV: 16.7 nmol/L, *P *= 0.045). Also, there was a nonsignificant trend for the LF diet to increase β-carotene (15.0 nmol/L, *P *= 0.09). None of the diets significantly affected plasma retinol, α-tocopherol, γ-tocopherol, or δ-tocopherol.

**TABLE 2:** Plasma concentrations of antioxidants, fat-soluble vitamins, and oxidative biomarkers in healthy adults with overweight and obesity at baseline, after 2 wk of consumption of an average American diet and after 5 wk of consumption of LF, MF, or AV diet^1^

Biomarker	Baseline (*n *= 45)	LF (*n *= 43)	MF (*n *= 42)	AV (*n *= 43)	Treatment effect (*P *)
Retinol, nmol/L	205 ± 9.2	213 ± 9.0	215 ± 9.7	200 ± 10.0	0.7
Lutein, nmol/L	33.6 ± 2.7	34.7 ± 2.2^b^	40.6 ± 2.1^b^	53.2 ± 5.2^a,^*	0.0004
α-Carotene, nmol/L	39.7 ± 5.0	46.8 ± 6.2^b^	69.9 ± 11.5^a,*^	56.9 ± 8.3^a,b,^*	0.01
β-Carotene, nmol/L	139 ± 9.5	154 ± 9.3	160 ± 13.2*	156 ± 11.4*	0.8
α-Tocopherol, μmol/L	13.0 ± 0.6	13.7 ± 0.6	13.3 ± 0.7	13.9 ± 0.9	0.8
γ-Tocopherol, μmol/L	2.4 ± 0.2	2.0 ± 0.1	2.0 ± 0.2	2.0 ± 0.3	0.5
δ-Tocopherol, μmol/L	0.3 ± 0.02	0.2 ± 0.01	0.3 ± 0.02	0.3 ± 0.02	0.9
oxLDL, U/L	65.8 ± 2.4	64.3 ± 2.9^a^	61.8 ± 2.2^a^	58.0 ± 2.1^b,*^	0.02
Isoprostane, pg/mL	26.2 ± 1.7	27.1 ± 2.4	25.7 ± 1.5	25.2 ± 1.4	0.6

^1^All values are means ± SEMs (*n *= 42–45). Values with different superscript letters differ (*P* < 0.05). ^*^Different from baseline, *P*< 0.05. AV, avocado diet; LF, lower-fat diet; MF, moderate-fat diet; oxLDL, oxidized LDL.

### Biomarkers of oxidative stress

**FIGURE 4:**
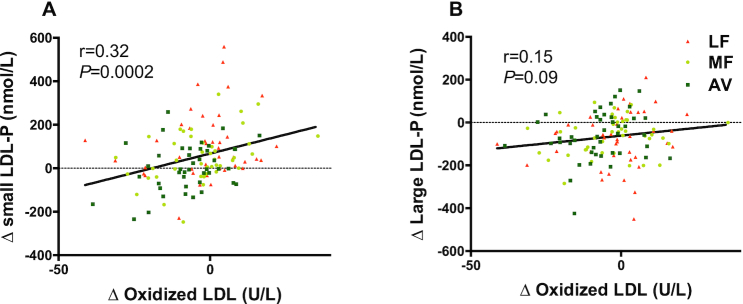
The correlation between changes in oxidized LDL (oxLDL) and LDL subclasses caused by all 3 diets in healthy adults with overweight and obesity (aged 21–70 y). (A) The correlation between changes in oxLDL and small LDL-P. (B) The correlation between changes in oxLDL and large LDL-P. AV, avocado diet; LDL-P, LDL particles; LF, lower-fat diet; MF, moderate-fat diet.

Compared with baseline, only the AV diet significantly decreased plasma oxLDL (−7.0 U/L, −8.8%,*P *= 0.0004). Moreover, the reduction in oxLDL by the AV diet was significantly greater (*P *= 0.05 and *P *= 0.03) than that by the MF and LF diets. None of the diets significantly affected plasma F_2_-isoprostane (Table 2, Figure 3). The change in oxLDL was significantly correlated with the change in sdLDL particles (*r *= 0.32, *P *= 0.0002, **Figure 4**) and sdLDL cholesterol (*r *= 0.47, *P* < 0.0001) but not large LDL particles (*r *= 0.15, *P *= 0.09, Figure 4) or large buoyant LDL-C (*r *= −0.03, *P *= 0.8). No correlations were observed between the change in plasma antioxidants and oxLDL.

### Activity of lipid transfer proteins

**FIGURE 5:**
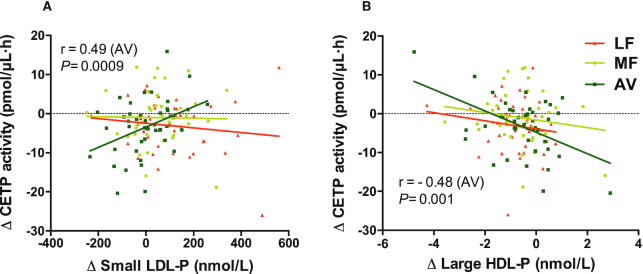
Correlations between change in CETP activity and change in LDL and HDL subclasses for the 3 diets in healthy adults with overweight and obesity (aged 21–70 y). (A) Correlation between change in CETP activity and change in small LDL-P. (B) Correlation between change in CETP activity and change in large HDL-P. AV, avocado diet; CETP, cholesteryl ester transfer protein; HDL-P, HDL particles; LDL-P, LDL particles; LF, lower-fat diet; MF, moderate-fat diet.

Compared with baseline, the ex vivo CETP activity decreased in plasma samples from both the LF diet [−3.0 pmol/(μL·h), *P *= 0.02] and AV diet [−3.2 pmol/(μL·h), *P *= 0.0007] compared with baseline. Only the comparison between the AV and MF diet was significant for CETP activity (*P *= 0.02) (**Table 3**). All 3 diets did not change PLTP or LCAT activity. We also found the change in CETP activity was significantly correlated with the change in sdLDL particles only during the AV diet (*r* = 0.49, *P *= 0.0009). In addition, the change in CETP activity was inversely correlated with the change in large HDL particles (*r *= −0.48, *P *= 0.001) after the AV diet only (**Table 4**, **Figure 5**).

**TABLE 3:** CETP, PLTP, and LCAT activity in healthy adults with overweight and obesity at baseline and after 5 wk of consumption of LF, MF, or AV diet^1^

Lipid transfer protein activity	Baseline (*n *= 45)	LF (*n *= 43)	MF (*n *= 42)	AV (*n *= 43)	Treatment effect (*P* )
CETP, pmol/(μL·h)	55.3 ± 1.4	52.8 ± 1.5^a,b,^*	54.4 ± 1.2^a^	52.1 ± 1.2^b,^*	0.02
PLTP, pmol/(μL·h)	20.3 ± 0.6	19.7 ± 0.7	20.0 ± 0.8	20.6 ± 0.7	0.5
LCAT, % change	6.2 ± 0.5	8.5 ± 0.7	8.0 ± 0.6	9.0 ± 0.7	0.5

^1^All values are means ± SEMs (*n *= 42–45). Values with different superscript letters differ (*P* < 0.05). ^*^Significant change compared with baseline average American diet, *P*< 0.05. AV, avocado diet; CETP, cholesteryl ester transfer protein; LCAT, lecithin/cholesterol acyltransferase; LF, lower-fat diet; MF, moderate-fat diet; PLTP, phospholipid transfer protein.

**TABLE 4:** Pearson correlation coefficients between changes in lipoprotein subclasses and CETP activity after 5 wk of consumption of the AV diet in healthy adults with overweight and obesity^1^

	Correlation coefficients between change values from baseline (*r*)		
	ΔCETP	ΔPLTP	ΔLCAT
ΔSmall HDL-P	0.03	−0.09	0.01
ΔLarge HDL-P	−0.48*	0.12	0.07
ΔSmall LDL-P	0.49*	−0.08	0.05
ΔLarge LDL-P	0.09	0.09	−0.21

1 ^*^Pearson correlation *P*< 0.01. CETP, cholesteryl ester transfer protein; HDL-P, HDL particles; LCAT, lecithin/cholesterol acyltransferase; LDL-P, LDL particles; PLTP, phospholipid transfer protein.

### Relative levels of mRNA of proinflammatory genes

The mean changes in expression of 4 proinflammatory genes are presented in **Supplemental Table 3**. We did not observe significant changes in *VCAM1*, *ICAM1*, *MCP1*, or *IL1B* mRNA levels after diet treatments compared with baseline or between the diets in the PBMC samples from a random subset of participants (*n *= 21).

## Discussion

To our knowledge, this is the first randomized controlled feeding trial to evaluate the effects of avocado consumption on biomarkers of oxidative status. A high-MUFA moderate-fat diet that included 1 avocado per day for 5 wk decreased plasma oxLDL by 8.8% compared with baseline AAD. Furthermore, we found that the oxLDL-lowering effect of avocados does not appear to be due to fatty acids since the MF diet with a matched fatty acid profile did not lower oxLDL.

The change in oxLDL was correlated with a change in number of sdLDL particles but not large, buoyant LDL, especially for the avocado diet. These findings suggest that avocados may decrease oxLDL by a mechanism that involves decreasing sdLDL. Several factors may influence the susceptibility of LDL to oxidation, including its size and composition, as well as the presence of endogenous antioxidants. Small LDL particles are particularly atherogenic since they penetrate the vessel wall more easily than larger LDL particles (20). Furthermore, small, dense lipoprotein particles are more likely to be retained by the extracellular matrix since they have been shown to bind to intimal proteoglycans in vitro (21). LDL particles transport several antioxidants, such as α-tocopherol, ubiquinone, and the carotenoids, β-carotene and lycopene. Lower concentrations of α-tocopherol and ubiquinone have been reported for sdLDL particles compared with buoyant LDL (22). It also has been suggested that surface lipid fluidity and composition may account for the greater susceptibility of sdLDL to oxidation, too (23). sdLDL particles that are depleted of free cholesterol have been reported to be less resistant to oxidation (24). Tribble et al. (25) reported that the resistance time to in vitro oxidative challenge of LDL particles was not related to the α-tocopherol, β-carotene, protein, triglyceride, or phospholipid content in LDL but was significantly correlated with the unesterified cholesterol content (*r* = 0.46; *P* < 0.001) and was inversely associated with the cholesterol ester content (*r* = 0.28; *P* < 0.05). This suggests that unesterified cholesterol in LDL may impart resistance to oxidative modification, possibly by altering properties on the surface monolayer where they reside.

Foods rich in polyphenols and antioxidant vitamins have been shown to protect LDL from oxidation (26–31). In contrast, clinical studies have not convincingly demonstrated that supplementation of certain antioxidants such as α-tocopherol, vitamin C, or β-carotene can protect LDL from oxidation (32, 33). The reductions of oxLDL we observed with avocados are consistent with previous studies with nuts and the Mediterranean diet (9, 27, 28, 30, 34–36), which are also rich in antioxidants and MUFAs. In the present study, the difference in dietary intake is consistent with the change in plasma antioxidants except for a trend for a reduction in γ-tocopherol (Tables 1 and 2). This may be due to the increased dietary α-tocopherol intake since serum γ-tocopherol typically is reduced by α-tocopherol supplementation (37). Both AV and MF diets significantly increased plasma β-carotene and α-carotene. Our study also showed a greater increase in plasma lutein in response to the AV diet compared with the LF and MF diets. Although we did not observe an association between the decrease in oxLDL and the increase in plasma carotenoids on the AV diet, a decrease in circulating oxLDL on the AV diet may be affected by both a decrease in sdLDL and an increase in plasma carotenoids, especially lutein. Plasma lutein and zeaxanthin are largely transported by HDL particles (38, 39). The concentration of HDL_3_ after consumption of the AV diet was significantly higher than after the LF diet (11). Previous evidence has shown that small, dense HDL_3_ particles provide potent protection of LDL in vivo from oxidative damage (40–42), and the increased carotenoid content of HDL particles following consumption of fruit and vegetables would enhance the antioxidant properties of HDL (43). Besides fat-soluble polyphenols and antioxidant vitamins, avocados are also rich in fiber, phytosterols, and other bioactives that may also contribute to the oxLDL reduction. Further studies are needed to determine which bioactives in avocados contribute to the reduction in oxLDL.

CETP, PLTP, and LCAT are plasma proteins involved in the exchange of cholesteryl esters and phospholipids between HDL and other lipoproteins. They are also responsible for the modification of LDL particles and lipoprotein remnants. The reduction of CETP activity by the AV diet provides insight about a potential mechanism by which avocados decrease sdLDL since CETP-mediated triglyceride (TG) enrichment and hepatic lipase-mediated lipolysis of apoB-containing particles yield sdLDL and decrease HDL cholesterol. In the present study, CETP activity was positively associated with sdLDL while negatively associated with large HDL cholesterol. In addition, this correlation was only observed for the AV diet. Although studies have shown that oleic acid may lower CETP activity (44), the MF diet in our study did not affect CETP activity. The reduction in CETP activity by the AV diet may be due to bioactive compounds in avocados beyond oleic acid. The LF diet also lowered CETP activity, while the change in CETP by the LF diet was not significantly correlated with changes in lipid markers, suggesting that the reduction of CETP may be due to other metabolic pathways. One possible explanation is that because the TG enrichment of VLDL and sdLDL was increased by the LF diet, the CETP was used for the TG–cholesterol ester (CE) exchange between lipoproteins and therefore was decreased (in the ex vivo assay). Another possible explanation is that the sdLDL production in response to the LF diet was due to a decrease in VLDL clearance (in response to high TG concentrations) and the conversion to lipoprotein remnants, rather than the exchange of TG and CE between VLDL and LDL by CETP. Since the LF diet decreased total cholesterol production via a reduction in SFAs, there may be a decrease in the intracellular cholesterol-regulatory pool in hepatocytes with a subsequent decrease in CETP (45). Based on our previous findings (11), it is reasonable to conclude that the LF diet increased TG production, yielding large TG-enriched VLDL particles, decreasing VLDL clearance, and resulting in larger LP remnants that were subsequently modified to LDL_4_. The causal relation between changes in CETP activity and plasma lipid concentrations induced by the diet is not clearly established. One study showed both low-fat and high-MUFA diets decreased plasma CETP concentrations in young, healthy, normolipemic men (46), which is consistent with our findings. A lipid kinetic study is needed to determine the metabolic pathways that affect sdLDL production by the LF and AV diets.

The strengths of the present study include the randomized, controlled full feeding design that enabled us to compare test diets to differentiate the effects of avocado bioactives from their constituent fatty acids on oxLDL. We also explored the possible impact of LDL particle size and plasma carotenoids on LDL oxidation status and investigated the metabolic pathways that affect sdLDL production in response to diet. Limitations of the study include the relatively short duration of the diet intervention, the analytical method used to quantify F_2_-isoprostanes, and the small subset sample size for assessing oxLDL-related proinflammatory gene expression. A longer-term diet intervention and measuring additional biomarkers of oxidative stress, inflammation, and antioxidant capability will be helpful to further understand the effect of avocados on inflammation and endothelial function. Nonetheless, we believe that our study provides new evidence for an important role of avocado bioactives (in addition to the beneficial effects of their fatty acid profile) in affecting the atherogenicity of LDL, which may confer additional benefits to CVD risk control beyond the LDL-C reduction (11).

## Conclusions

Including 1 avocado per day in a heart-healthy diet decreased circulating oxLDL and increased plasma lutein concentrations compared with a typical Western diet, a macronutrient- and fatty acid–matched moderate-fat diet, and a lower-fat, high-carbohydrate diet. On the basis of our findings, we conclude that these benefits are due to the bioactive compounds present in avocados beyond their fatty acids. The decrease in small LDL particles may contribute to the oxLDL-lowering effect observed on the avocado diet. In addition, the high-carbohydrate, lower-fat diet did not increase oxLDL, even though it increased sdLDL particles. An increase in fruit, vegetables, and whole grains in the diet may protect the atherogenic lipoproteins from oxidation.

Avocados have a unique nutrient and bioactive profile that appears to play an important role in reducing LDL oxidation, hence decreasing LDL atherogenicity. Additional long-term prospective and intervention studies are needed to evaluate the effect of avocado consumption on clinical CVD outcomes and determine the role that avocados may play in the primary and secondary prevention of CVD.

## Supplementary Material

nxz231_Supplemental_Files

## Notes

Supported by a grant from the Hass Avocado Board. The clinical trial was also supported by the National Center for Research Resources (grant UL1 RR033184) and the National Center for Advancing Translational Sciences (grant UL1 TR000127).

Author disclosures: LW, LT, LH, THS, K-HH, and JDL, no conflicts of interest. PMK-E received funding from the Hass Avocado Board to conduct this study and is a member of the Avocado Nutrition Science Advisory. The Hass Avocado Board had no role in the design and conduct of the study; in the collection, analysis, and interpretation of the data; or in the preparation, review, or approval of the manuscript. The content is solely the responsibility of the authors and does not necessarily represent the official views of the National Institutes of Health.

Supplemental Figure 1 and Supplemental Tables 1–3 are available from the “Supplementary data” link in the online posting of the article and from the same link in the online table of contents at https://academic.oup.com/jn.

Abbreviations used: AAD, average American diet; AV, avocado; CE, cholesterol ester; CETP, cholesteryl ester transfer protein; CVD, cardiovascular disease; ICAM1, intercellular adhesion molecule 1; LCAT, lecithin/cholesterol acyltransferase; LDL-C, LDL cholesterol; LF, lower fat; MCP1, monocyte chemoattractant protein 1; MF, moderate fat; oxLDL, oxidized LDL; PBMC, peripheral blood mononuclear cells; PLTP, phospholipid transfer protein; sdLDL, small, dense LDL; TG, triglyceride; VCAM1, vascular cell adhesion molecule 1.
