# Supplementary material for: A Moderate-Fat Diet with One Avocado per Day Increases Plasma Antioxidants and Decreases the Oxidation of Small, Dense LDL in Adults with Overweight and Obesity: A Randomized Controlled Trial
Source: J Nutr. 2019 Oct 14;150(2):276–84. doi: 10.1093/jn/nxz231 (PMC7373821; doi:10.1093/jn/nxz231)
Supplement: nxz231_Supplemental_Files [file nxz231_supplemental_files.zip › Supplemental_Figures_August 19_2019.pptx]

## Slide 1
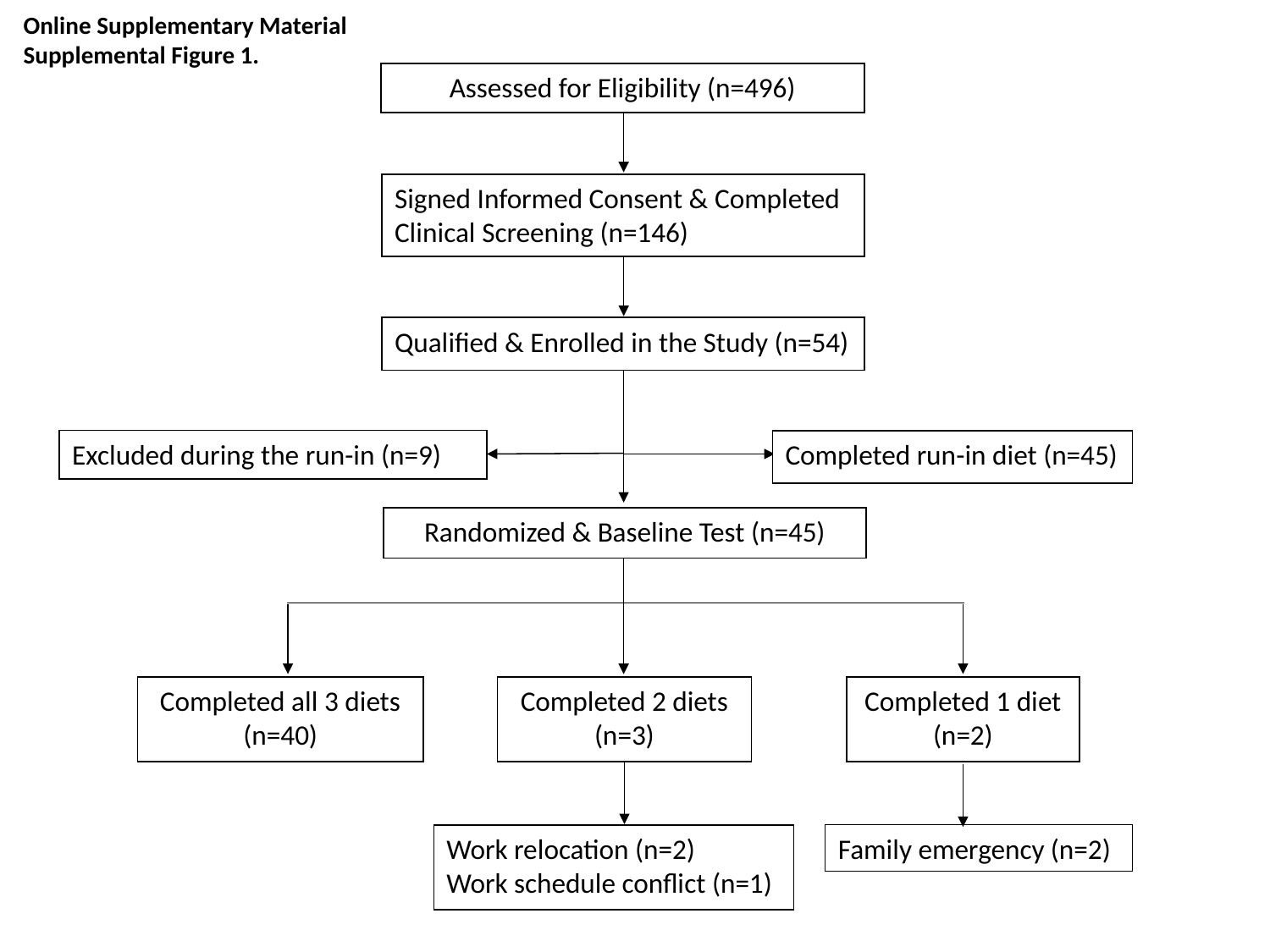

# Online Supplementary Material Supplemental Figure 1.
Assessed for Eligibility (n=496)
Signed Informed Consent & Completed Clinical Screening (n=146)
Qualified & Enrolled in the Study (n=54)
Excluded during the run-in (n=9)
Completed run-in diet (n=45)
Randomized & Baseline Test (n=45)
Completed all 3 diets (n=40)
Completed 2 diets (n=3)
Completed 1 diet (n=2)
Family emergency (n=2)
Work relocation (n=2)
Work schedule conflict (n=1)
