# Supplementary material for: A Moderate-Fat Diet with One Avocado per Day Increases Plasma Antioxidants and Decreases the Oxidation of Small, Dense LDL in Adults with Overweight and Obesity: A Randomized Controlled Trial
Source: J Nutr. 2019 Oct 14;150(2):276–84. doi: 10.1093/jn/nxz231 (PMC7373821; doi:10.1093/jn/nxz231)
Supplement: nxz231_Supplemental_Files [file nxz231_supplemental_files.zip › Supplemental_Tables_August_19_2019.docx]

**Online Supplementary Material**

**Supplemental Table 1.**  Nutrient content of one Hass avocado (~136g)

| **Nutrient** | **Content in Hass Avocado**  **(1 fruit, ~136g on average)** |
| --- | --- |
| Calories (kcal) | 230 |
| Protein (g) | 3 |
| Total fat (g) | 21 |
| SFA (g) | 3 |
| MUFA (g) | 13 |
| PUFA (g) | 2.5 |
| Cholesterol | 0 |
| Beta-sitosterol (mg) | 103 |
| Dietary fiber (g) | 9 |
| Potassium (mg) | 690 |
| Folate (μg) | 121 |
| α-carotene (μg) | 33 |
| β-carotene (μg) | 86 |
| α-tocopherol (mg) | 2.7 |
| γ-tocopherol (mg) | 0.4 |
| Lutein & zeaxanthin (μg) | 369 |
| Total phenolics (mg) | 260 |

Data resource: U.S. Department of Agriculture, Agricultural Research Service, National Nutrient Database for Standard Reference, <https://ndb.nal.usda.gov/ndb/>, accessed October 2010.

**Supplemental Table 2.** Primer Sequence of targeted genes

| Gene | Primer Sequence | Amplification Size, bp |
| --- | --- | --- |
| *MCP1* | TCAAACTGAAGCTCGCACTC | 390 |
|  | CTGGGGAAAGCTAGGGGAAA |  |
| *VCAM1* | GCCCATCTATGTCCCTTGCT | 427 |
|  | CCTGAAAGTCAACCCAGTGC |  |
| *ICAM1* | AACCCCACAGTCACCTATGG | 478 |
|  | ACATTGGAGTCTGCTGGGAA |  |
| *IL1B* | ACTGAAAGCTCTCCACCTCC | 426 |
|  | TCCACATTCAGCACAGGACT |  |
| *GUSB* | TGCAGAGGAAGTGATGCAGA | 467 |
|  | CTGCTCCATACTCGCTCTGA |  |

**Supplemental Table 3.** mRNA expression of MCP1, VCAM1, ICAM1, and IL1B of PBMCs from a subset of samples (n=21) from healthy adults with overweight and obesity (21-70y) after 5 weeks on the LF, MF, or AV diets

|  | Baseline | LF | MF | AV |
| --- | --- | --- | --- | --- |
| *VCAM1* | 1.0 ± 0.28 | 1.59 ± 0.44 | 1.50 ± 0.48 | 1.37 ± 0.39 |
| *ICAM1* | 1.0 ± 0.26 | 1.39 ± 0.37 | 1.04 ± 0.22 | 1.18 ± 0.29 |
| *MCP1* | 1.0 ± 0.30 | 0.69 ± 0.20 | 1.06 ± 0.37 | 1.10 ± 0.30 |
| *IL1B* | 1.0 ± 0.24 | 1.63 ± 0.45 | 1.08 ± 0.25 | 1.61 ± 0.35 |

All values are means ± SEMs (n=21). Gene expression in relative abundance is expressed as fold changes of baseline.
